# Antioxidative 2D Bismuth Selenide via Halide Passivation for Enhanced Device Stability

**DOI:** 10.3390/nano13142056

**Published:** 2023-07-12

**Authors:** Jiayi Chen, Guodong Wu, Yamei Ding, Qichao Chen, Wenya Gao, Tuo Zhang, Xu Jing, Huiwen Lin, Feng Xue, Li Tao

**Affiliations:** 1 School of Materials Science and Engineering, Southeast University, Nanjing 211189, China; jiayi_chen@seu.edu.cn (J.C.); wuguodongjohn@gmail.com (G.W.);; 2 Jiangsu Key Laboratory for Advanced Metallic Materials, Southeast University, Nanjing 211189, China

**Keywords:** two-dimensional materials, bismuth selenide, antioxidation, field-effect transistors

## Abstract

The topological insulator 2D Bi_2_Se_3_ is promising for electronic devices due to its unique electronic properties; however, it is challenging to prepare antioxidative nanosheets since Bi_2_Se_3_ is prone to oxidation. Surface passivation using ligand agents after Bi_2_Se_3_ exfoliation works well to protect the surface, but the process is time-consuming and technically challenging; a passivation agent that is stable under a highly biased potential is significant for in situ passivation of the Bi_2_Se_3_ surface. In this work, the roles of halide anions (Cl^−^, Br^−^, and I^−^) in respect of the chemical properties of synthetic Bi_2_Se_3_ nanosheets during electrochemical intercalated exfoliation were investigated to determine the antioxidation capacity. It was found that Bi_2_Se_3_ nanosheets prepared in a solution of tetrabutylammonium chloride (TBA^+^ and Cl^−^) have the best oxidation resistance via the surface bonding of Bi with Cl, which promotes obtaining better device stability. This work paves an avenue for adjusting the components of the electrolyte to further promote the stability of 2D Bi_2_Se_3_-nanosheet-based electronic devices.

## 1. Introduction

Two-dimensional (2D) semiconductors have greatly facilitated the rapid development of electronic and optoelectronic devices, owing to their remarkable charge transport properties and robust mechanical properties [1,2,3]. Among the 2D material family, 2D Bi_2_Se_3_ is a promising material for electronic devices due to its unique electronic properties as a topological insulator, which means that its bulk is an insulator while the surface exhibits metallic behavior [4,5,6]. The surface conductivity arises from topologically protected surface states, resulting in unique electronic properties, including high carrier mobility and immunity to scattering by non-magnetic impurities [7,8]. Such unique electronic properties make 2D Bi_2_Se_3_ ideal for low-power and high-speed electronic devices, especially those investigated as field-effect transistors (FETs) [9,10,11,12]. Theoretical studies have predicted the behavior of 2D Bi_2_Se_3_ FETs with a thickness regime of 1–6 nm, in which a small gap induced by hybridization between the top and bottom surfaces is sufficient to obtain transistor operation at room temperature [9,12]. The primary research on two-dimensional Bi_2_Se_3_-based FETs aims to improve the electrical performance and reliability for application via optimizing the fabrication method or modifying the device structures [13]. However, as one of the challenging problems, Bi_2_Se_3_ nanosheets are air-sensitive and prone to oxidation, which leads to the degradation of device performance when exposed to air [14,15,16,17].

To address this issue, researchers have proposed various strategies to protect Bi_2_Se_3_ nanosheets from oxidation, i.e., encapsulation techniques that can shield the material from the air or passivate the nanosheet surface with some organic or inorganic molecules [18]. These strategies can proceed after the Bi_2_Se_3_ nanosheet is synthesized using the techniques of chemical vapor deposition, molecular beam epitaxy, or pulsed laser deposition [19,20,21]. However, these techniques are limited by the stringent reaction conditions and complicated transferring procedures required to transfer the thin films from the growth substrate to the target substrate [22]; these transfers are time-consuming and technically challenging, leading to irreversible damage to the device performance when considering potential oxidation during the transfer.

In comparison, using solution-phase exfoliation to produce Bi_2_Se_3_ nanosheets and passivating them in situ in the solution can be more convenient, avoiding the exposure of unprotected nanosheets to the air [20,23]. Surface passivation in situ in the solution is a simpler and more scalable method, which can better control the thickness and quality of the passivation layer. Passivation agents such as thiols have been determined to be effective by forming strong covalent bonds with the surface of the Bi_2_Se_3_ nanosheets [24,25]. Yang et al. compared the antioxidant ability of electrochemically modified and thiol-functionalized Bi_2_Se_3_-based nanosheets, finding that pentanedithiol-modified surfaces can avoid oxidation in the air for hours [24]. However, due to their instability under a highly biased potential, such agents are mostly utilized after Bi_2_Se_3_ exfoliation to avoid agent decomposition during the exfoliation process [25]. Less consideration is put into passivation agents that are stable under a highly biased potential, while it is possible to passivate the Bi_2_Se_3_ surface in situ.

In this work, we investigate the roles of halide anions (Cl^−^, Br^−^, and I^−^) in respect of the chemical properties of synthetic Bi_2_Se_3_ nanosheets, especially the oxidation resistance that is significant for device stability. Electrochemical intercalation exfoliation was carried out in solutions containing tetrabutylammonium cations (TBA^+^), while the anions were changed within halide anions (Cl^−^, Br^−^, and I^−^). Ultrathin Bi_2_Se_3_ nanosheets were successfully synthesized with most thicknesses around 5 nm. It was found that Bi_2_Se_3_ nanosheets prepared in a solution of TBAC (TBA^+^ and Cl^−^) have the best oxidation resistance. This work paves an avenue to adjust the components of the electrolyte to further promote the stability of 2D Bi_2_Se_3_-nanosheet-based electronic devices.

## 2. Materials and Methods

### 2.1. Chemicals

Bismuth selenide (Bi_2_Se_3_, vacuum deposition grade, 99.995%) was purchased from Alfa Aesar (Waltham, MA USA). Acetonitrile (C_2_H_3_N, 99.8%, H_2_O ≤ 0.003%), 1-Methyl-2-pyrrolidinone (C_5_H_9_NO, NMP, for HPLC, 99.5%), silver nitrate (AgNO_3_, 99.99% metals basis), and tetrabutylammonium perchlorate (C_16_H_36_ClNO_4_, electrochemical grade) were purchased from Shanghai Aladdin Biochemical Technology (Shanghai, China). Tetrabutylammonium chloride (C_16_H_36_NCl, 99%), tetrabutylammonium bromide (C_16_H_36_NBr, 99%), tetrabutylammonium iodide (C_16_H_36_NI, 99%), isopropyl alcohol (C_3_H_8_O, IPA, 99.5%), and polyvinylpyrrolidone ((C_6_H_9_NO)n, PVP, MW 24000) were purchased from Shanghai Macklin Biochemical Technology (Shanghai, China). All chemicals were used without further purification.

### 2.2. Synthesis of Bi_2_Se_3_ Nanosheets

A single-crystal Bi_2_Se_3_ plate was used as the working electrode to prepare Bi_2_Se_3_ nanosheets via electrochemical exfoliation. A reference electrode of Ag/Ag^+^ (0.01 M AgNO_3_) and a counter electrode of a Pt foil were used in the electrochemical cell. The electrochemical exfoliation proceeded under −2.5 V (vs. Ag/Ag^+^) for 10 min in acetonitrile solution containing 0.05 M TBA^+^, in which the anion could be changed and selected as Cl^−^, Br^−^, and I^−^ in this work. The exfoliated Bi_2_Se_3_ was soaked immediately in an NMP solution containing 20 g/L PVP and sonicated for 1 min. The suspension was centrifuged for 10 min under a rotation speed of 10,000 rpm, and Bi_2_Se_3_ was washed through 3 cycles of the above process with IPA refilled to maintain liquid volume. The supernatant of the Bi_2_Se_3_ nanosheets was obtained with final centrifugation for 3 min under a rotation speed of 1000 rpm. The Bi_2_Se_3_ nanosheets prepared in different electrolytes containing Cl^−^, Br^−^, and I^−^ were denoted as TBAC-Bi_2_Se_3_, TBAB-Bi_2_Se_3_, and TBAI-Bi_2_Se_3_, respectively.

### 2.3. Characterization

X-ray diffraction (XRD, Rigaku Smart Lab Cu Kα, Tokyo, Japan) was used to determine the crystalline structure of the synthetic nanosheets. X-ray photoelectron spectroscopy (XPS, ESCALAB 250Xi analysis system at 12.5 kV, Waltham, MA, USA) was applied to measure the chemical states using monochromatized Al Kα as the X-ray source. Raman measurements (WITec Alpha 300R, Ulm, Germany) were carried out under a wavelength of 532 nm using a laser with a 50× objective lens to characterize the crystallinity of the Bi_2_Se_3_ nanosheets. The morphologies and elemental distributions were determined via a transmission electron microscope (TEM, Talos F200X, Waltham, MA, USA) along with affiliated energy-dispersive spectroscopy (EDS, Super-X EDS, Waltham, MA, USA), while the thickness was determined via atomic force microscopy (AFM, Bruker Dimension Icon, Karlsruher, Germany). The electrical characteristics were measured with a semiconductor analyzer (Keysight 2902A, Santa Rosa, CA, USA) under ambient conditions at room temperature.

### 2.4. Fabrication of Field-Effect Transistors 

A 30 nm Al_2_O_3_/n-Si wafer was prepared using atomic layer deposition (ALD, PICOSUN R-200 Standard ALD system, Espoo, Finland), with 315 cycles of trimethylaluminum and water vapor pulses under 300 °C. Bi_2_Se_3_ nanosheet suspensions were dropped onto the as-prepared silicon wafer and spin-coated at 5000 rpm to obtain a discontinuous membrane of Bi_2_Se_3_ nanosheets. Subsequently, a thin film of polymethyl methacrylate (Microchem PMMA 950 A4, NEWTON, MA, USA) was spin-coated onto the membrane and baked at 180 °C for 3 min. Electron-beam lithography (EBL, FEI Inspect F50 ELPHY Quantum, 20 kV, Dortmund, Germany) was applied to locate a single Bi_2_Se_3_ nanosheet and define appropriate electrodes. The write field was aligned with a 1 mm standard chess wafer. The electron beam lens aperture was set to 3.5 with a magnification of 1000×, and the scanning step was fixed to 20 nm to achieve a balance between speed and precision. Electron beam evaporation (EBE, Vnano VZS-600, Beijing, China) was applied to deposit 5 nm Ti and 50 nm Au films, followed by a standard lift-off procedure.

## 3. Results and Discussion 

### 3.1. Characterization of Synthetic Bi_2_Se_3_ Nanosheets

**Figure 1:**
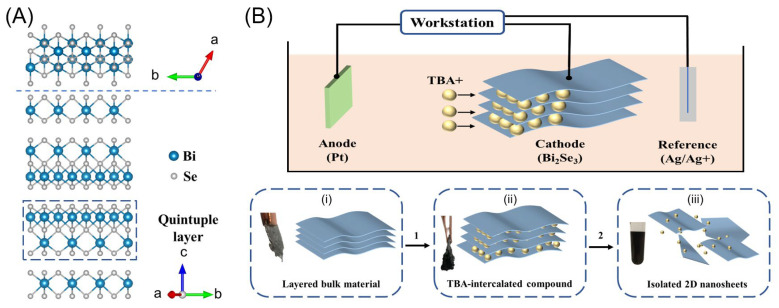
(**A**) Top and side views of the atomic structure of layered Bi_2_Se_3_ crystal and (**B**) schematic images of the electrochemical cathodic exfoliation of Bi_2_Se_3_ using TBA^+^ as the intercalant. Insets: photographs and schematic images of (**i**) the working electrode of the Bi_2_Se_3_ plate before electrochemical intercalation, (**ii**) TBA-intercalation Bi_2_Se_3_ with volume expansion, and (**iii**) Bi_2_Se_3_ nanosheet dispersion after sonication in NMP solution.

The bulk Bi_2_Se_3_ has a layered structure in which each layer comprises five atomic layers called quintuple layers (QL) [5]. The atoms in each quintuple layer are bonded in the sequence Se-Bi-Se-Bi-Se, while the quintuple layers are held together via weak van der Waals interactions, as shown in Figure 1A. The weak van der Waals interactions between the layers benefit electrochemical exfoliation via cation-assisted intercalation. Thus, electrochemical intercalation exfoliation was carried out in this work through a three-electrode system that used the bulk Bi_2_Se_3_ plate as the working electrode (Figure 1B, see more details in Section 2).

Notably, the TBA^+^ cation was used as the intercalation agent since it has been determined to be efficient for the exfoliation of layered materials [26]. Intercalation of the TBA^+^ cation brings less charge and more layer expansion because of its larger ionic size compared to metal ions such as Li^+^ or Na^+^, allowing it to intercalate with less damage over the crystal structures. In addition, the anions were changed in this study to Cl^−^, Br^−^, and I^−^ to determine the anion effect over the chemical properties of the synthetic Bi_2_Se_3_ nanosheets, especially the ability for oxidation resistance that is significant for device stability. 

To facilitate efficient exfoliation, the working voltage had to be optimized for the TBA^+^ intercalation. The electrochemistry of TBA^+^ intercalation into the bulk Bi_2_Se_3_ was then studied using linear sweep voltammetry (Figure S1a) before a decision was made on the constant potential for TBA^+^ intercalation. A peak cathodic current was observed around −2.2 V, corresponding to the TBA^+^ intercalation process. The potential was then chosen as −2.5 V for the electrochemical intercalation of TBA^+^ cations in the TBAB solution. As shown in Figure S1b, the current exhibits a fluctuating profile with time due to a significant change in the host structure during the exfoliation, breaking the ionic channel or decreasing the electronic conductivity. After electrochemical intercalation, an apparent volume expansion of the bulk Bi_2_Se_3_ is observed (Figure 1B, inset ii), and a short sonication time yields a concentrated dispersion that contains the Bi_2_Se_3_ nanosheets (Figure 1B, inset iii).

**Figure 2:**
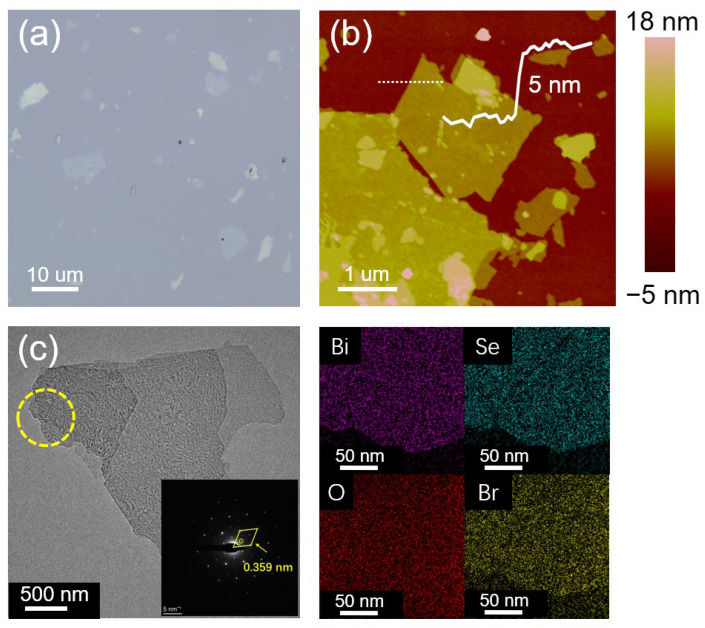
(**a**) Optical image and (**b**) atomic force microscopy topography image of TBAB-Bi_2_Se_3_ nanosheet with a height profile along the dashed line that indicates a layer thickness of around 5 nm. (**c**) Transmission electron microscopy image with the corresponding energy dispersive spectroscopy mapping images of the exfoliated TBAB-Bi_2_Se_3_. The elements Bi (purple), Se (indigo), O (red), and Br (yellow) are shown. Inset figure: the selected area electron diffraction pattern of the TBAB-Bi_2_Se_3_ nanosheet.

The optical microscopy image of the drop-casted TBAB-Bi_2_Se_3_ nanosheet on a Si substrate with a SiO*_x_* layer indicates a large number of TBAB-Bi_2_Se_3_ nanosheets with lateral sizes around 10 μm, as shown in Figure 2a. The thicknesses can be further determined distinctly via AFM in Figure 2b, in which the thickness of the TBAB-Bi_2_Se_3_ nanosheet is around 5 nm, indicating that the nanosheet consists of several QLs since a mono QL is around 1 nm. Notably, several nanosheets of smaller lateral sizes can also be observed with larger thicknesses, which might have been generated during the sonication process. The TEM images and the EDS mapping images are shown in Figure 2c. As observed, the lamellar structure of the TBAB-Bi_2_Se_3_ nanosheets is confirmed, although the structure was formed with inadequate exfoliation, in which some layers with smaller lateral sizes remain unexfoliated. The remaining smaller Bi_2_Se_3_ layer suggests a potential sheet fracture during the electrochemical exfoliations. The exfoliation is generally efficient, with a high yield of large Bi_2_Se_3_ nanosheets using the electrochemical exfoliation under −2.5 V as an optimized potential. The EDS mapping shows a uniform distribution of the Bi and Se, suggesting an unchanged chemical element during the electrochemical exfoliation. The signals from the Br element in element mapping are found due to possible surface bonding of Br with Bi during the electrochemical exfoliation. In addition, the aspect ratio of the nanosheet morphology remains large, as shown in Figure S2, even though the anion was changed in the electrolyte during the exfoliation process.

**Figure 3:**
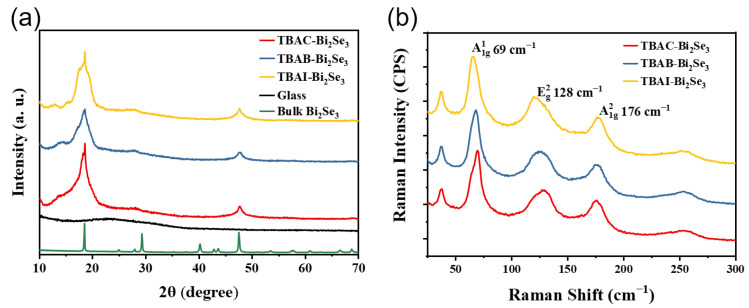
(**a**) X-ray diffraction (XRD) patterns of TBAC-Bi_2_Se_3_, TBAB-Bi_2_Se_3_, TBAI-Bi_2_Se_3_, and bulk Bi_2_Se_3_. (**b**) Raman spectra of TBAC-Bi_2_Se_3_, TBAB-Bi_2_Se_3_, and TBAI-Bi_2_Se_3_.

As is known, the intercalation of cations with smaller ionic radii occasionally changes the crystal structure of exfoliated nanosheets [27,28,29,30]. For example, Li^+^ intercalation results in a phase conversion from 2H to 1T in 2D MoS_2_ [31]. In this paper, XRD patterns of the three exfoliated Bi_2_Se_3_ nanosheets are shown to determine an unchanged crystal structure with the bulk Bi_2_Se_3_. Compared with the diffraction peaks shown in Figure S3 for the bulk Bi_2_Se_3_ powder (rhombohedral phase, PDF#JCPDS:033-0214), only two prominent diffraction peaks located at 18.5° and 47.6° corresponding to the (006) and (0015) planes can be observed in Figure 3a, indicating a preferential stacking orientation of the *z*-axis for the thin film of Bi_2_Se_3_ nanosheets. Such a phenomenon is caused by the high aspect-ratio of the Bi_2_Se_3_ nanosheets (Figure S2), which leads to high flexibility and only horizontal tiling of nanosheets on the substrate. On the other side, this proves the high quality of the Bi_2_Se_3_ nanosheet suspension as there is no bulk Bi_2_Se_3_ residue. In particular, the half-peak widths of the (006) and (0015) planes are broader than those of the pristine ones because of the significant decrease in the layer number of nanosheets. No other diffraction peaks can be found, suggesting an unchanged phase structure of the Bi_2_Se_3_ nanosheets compared with that of the bulk Bi_2_Se_3_. The XRD patterns present similar behavior for all three Bi_2_Se_3_ nanosheets of TBAC-Bi_2_Se_3_, TBAB-Bi_2_Se_3_, and TBAI-Bi_2_Se_3_. Thus, the anion change has not affected the crystal structures of the synthetic Bi_2_Se_3_ nanosheets. Moreover, a crystalline characteristic of the Bi_2_Se_3_ nanosheets is demonstrated in the selected area electron diffraction (SAED) pattern (inset figure in Figure 2c), in which a typical d-space of 0.359 nm corresponding to the (110) direction of the Bi_2_Se_3_ nanosheet is indicated. The results of SAED are consistent with those of XRD, which further confirms that the exfoliated nanosheet is crystalline Bi_2_Se_3_.

In addition, Raman spectroscopy was applied to characterize the Bi_2_Se_3_ nanosheets, as shown in Figure 3b. In the bulk Bi_2_Se_3_, Raman-active modes 1E_g_, 1A_1g_, 2E_g_, and 2A_1g_ were recorded [32] at about 37, 72, 132, and 174 cm^−1^, respectively, while the last three values were shifted to 69, 128, and 176 cm^−1^, respectively. The 3 cm^−1^ redshift of 1A_1g_ combined with asymmetric broadening can be attributed to the confined phonon states in the Bi_2_Se_3_ nanosheets, which have been confirmed with Fauchet and Campbell’s extended model [32,33], indicating a thickness of 6 QLs. In addition, a phenomenological exponential relation can explain that the full-width at half-maximum of the 2E_g_ mode broadens to 20 cm^-1^ in the finite size regime [32,34], and an average thickness of 6 QLs can be estimated. Therefore, the Raman spectroscopy data confirm the successful preparation of a few QL Bi_2_Se_3_ nanosheets, as shown by the AFM images.

### 3.2. Effects of Halide Ion over the Surface Stability of the Bi_2_Se_3_ Nanosheets

**Figure 4:**
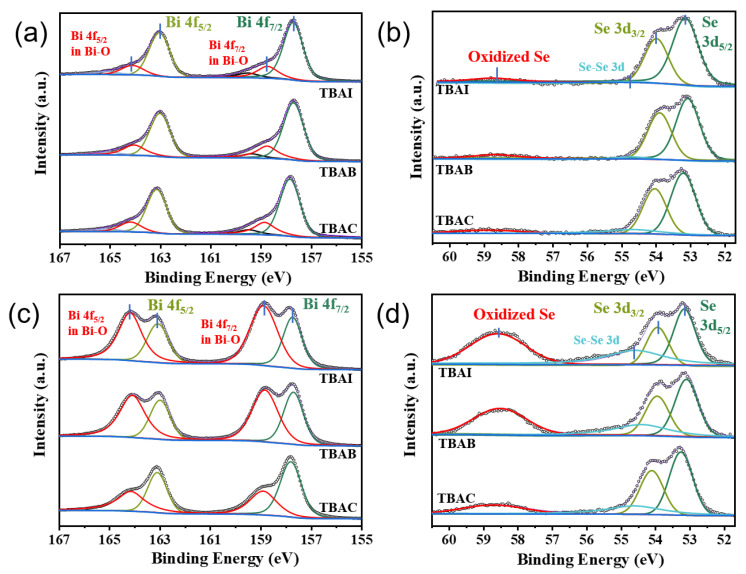
High-resolution X-ray photoelectron spectra of (**a**,**c**) Bi 4f and (**b**,**d**) Se 3d signals from the TBAC-Bi_2_Se_3_, TBAB-Bi_2_Se_3_, and TBAI-Bi_2_Se_3_ nanosheets for (**a**,**b**) the as-synthesized and (**c**,**d**) the air-exposed Bi_2_Se_3_ for 5 days, respectively. The spectra have been deconvolved to compare the specific components and labeled with different colors.

X-ray photoelectron spectroscopy was utilized to analyze the surface chemistry of the Bi_2_Se_3_ nanosheets as well as their antioxidation capacity by analyzing the oxidizing components. The XPS results were calibrated with the binding energy of C 1s (284.8 eV). The high-resolution X-ray photoelectron spectra of the as-synthesized TBAC-Bi_2_Se_3_, TBAB-Bi_2_Se_3_, and TBAI-Bi_2_Se_3_ nanosheets for the Bi 4f and Se 3d are shown in Figure 4a,b along with the deconvolved peaks. Two dominant peaks at approximately 163.0 and 157.8 eV in Bi 4f are indicated to be the Bi 4f_5/2_ and 4f_7/2_ in the Bi-Se bonds, consistent with the two peaks at approximately 54.0 and 53.1 eV in Se 3d for the Se 3d_3/2_ and 3d_5/2_ components. The as-synthesized nanosheets are likely to be oxidized slightly, which may be indicated by the peaks at 164.1 and 158.7 eV in Bi 4f corresponding to the oxidized Bi (Bi-O) and the peaks around 58.5 eV in Se 3d corresponding to the oxidized Se [14,17]. Specifically, the oxidized components of Bi and Se in the as-synthesized TBAC-Bi_2_Se_3_, TBAB-Bi_2_Se_3_, and TBAI-Bi_2_Se_3_ nanosheets show little distinguishable difference, as can be observed in Table 1, in which the estimated atomic ratios of the oxidized Bi and Se components from the deconvolved peaks areas are around 15% for all three samples. 

To determine the antioxidative capacity of the nanosheets, all the samples were air-exposed for 5 days, and XPS was carried out for the air-exposed samples, as shown in Figure 4c,d. Obviously, the intensity of the peaks corresponding to the oxidized components of Bi-O in Bi 4f increases after air exposure for all the samples of the TBAC-Bi_2_Se_3_, TBAB-Bi_2_Se_3_, and TBAI-Bi_2_Se_3_ nanosheets. However, it shows a much weaker oxidation trend for the TBAC-Bi_2_Se_3_ nanosheets since the smallest intensity is obtained. Such an oxidation trend is also reflected by Se 3d in Figure 4d, in which the peak intensity corresponding to the oxidized Se shows the smallest intensity for the TBAC-Bi_2_Se_3_ nanosheet. In addition, the increased peak intensity at 54.6 eV corresponding to Se-Se bonds indicates the formation of Se particles on the surface of the Bi_2_Se_3_ nanosheets during oxidation in the air. 

The estimated oxidized components from the thorough simulation for Bi 4f and Se 3d of the air-exposed Bi_2_Se_3_ nanosheets are shown in Table 1, and the total number of oxidized and unoxidized Bi atoms in each sample is fixed to 2 for comparison. The atomic ratio of oxidized Bi in TBAC-Bi_2_Se_3_ increased only from 0.34 to 0.52, which indicates antioxidative capacity in the order TBAC-Bi_2_Se_3_ > TBAB-Bi_2_Se_3_ > TBAI-Bi_2_Se_3_. Such antioxidative capacity is proposed to be affected by halide passivation through the bond formation between the Bi and halide atoms. The binding energy of the 4f electrons in Bi in freshly synthesized TBAC-Bi_2_Se_3_ increased by approximately 0.1 eV compared to TBAB-Bi_2_Se_3_ or TBAI-Bi_2_Se_3_. This is due to the decreasing electronegativity of the Cl, Br, and I elements. However, the chemical shift in the Bi 3f signal is not apparent because only a few halogen atoms displaced the Se vacancies on the surface of nanosheets, which can be proved with the EDS results. Several previous works have reported a small amount of the Se vacancy combined with selenium oxide and metal selenide [14,25,35]. As is known, the Se vacancy is prone to being refilled by other anions by forming Bi-anion bonds [36], and such vacancy refilling will affect the anti-resistance capacity of the Bi_2_Se_3_ nanosheets. We propose that a larger electronegativity of Cl than that of Br or I leads to increased electronic distribution from Bi to Cl, resulting in increased antioxidative capacity, although the TBAC-Bi_2_Se_3_ nanosheets will still be oxidized. 

### 3.3. Device Performance

**Figure 5:**
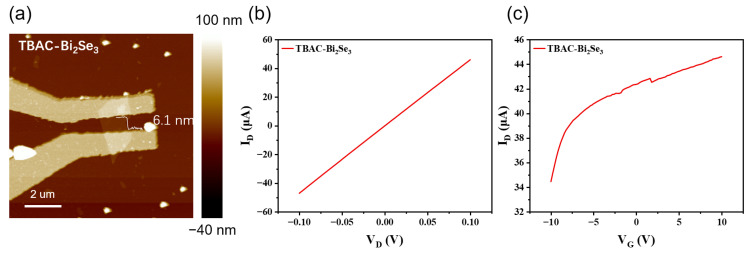
(**a**) Atomic force microscopy topography image of FET device using TBAC-Bi_2_Se_3_ nanosheet. (**b**) Output characteristic curve, gate voltage set to 0 V. (**c**) Transfer characteristic curve, V_D_ set to 0.1 V.

The thickness of the Bi_2_Se_3_ nanosheet in the fabricated FET is 6.1 nm, as measured via AFM and as shown in Figure 5a. To avoid oxidation in air, output characteristics (Figure 5b) and transfer characteristics (Figure 5c) were measured within one hour after the lift-off process. A liner curve of drain-to-source current (I_D_) versus drain-to-source voltage (V_D_) indicates good ohmic contact between the Bi_2_Se_3_ nanosheet channel and metal electrode without the existence of a Schottky barrier. In the absence of the contact resistance, the sheet resistance was estimated as 2.4 kΩ/sq. The I_D_ increasing monotonically with gate-to-source voltage (V_G_) in Figure 5c indicates the n-type nature of the Bi_2_Se_3_ nanosheet and the main current carrier is the electron. The field-effect mobility values (μ_FE_) of the Bi_2_Se_3_ nanosheet FET were extracted from the linear region of the I_D_-V_D_ curves using the following equation [37]: μ_FE_ = L_ch_ × g_m_/(W_ch_ × C_G_ × V_D_), in which L_ch_ is the channel length, W_ch_ is the channel width, C_G_ is the gate capacitance, and g_m_ is the terminal transconductance. The maximum μ_FE_ value of 58.5 cm^2^·V^−1^·s^−1^ was obtained in the negative range of V_G_, indicating the configuration of the N-channel depletion mode. This value is close to the estimated value (66 cm^2^·V^−1^·s^−1^) from the exponential relation between electron effective mobility and temperature [10] but is much higher than the experimental value (10 cm^2^·V^−1^·s^−1^ at 245 K) obtained for a mechanically exfoliated Bi_2_Se_3_ device [38]. To further expand the application of the Bi_2_Se_3_ nanosheet suspension, spin-coated films of Bi_2_Se_3_ were fabricated, as shown in Figure S4. Three layers of nanosheets were obtained after spin-coating more than 4 times, with a sheet resistance of 1 kΩ/sq, which is consistent with the output characteristics of the TBAC-Bi_2_Se_3_ FET device.

## 4. Conclusions

In conclusion, the antioxidative capacity of electrochemically exfoliated 2D Bi_2_Se_3_ nanosheets through changing the halide passivation was determined for the enhanced performance of electronic devices. Synthetic Bi_2_Se_3_ nanosheets with thicknesses of less than 5 nm can be obtained with a large lateral size greater than 5 μm, independent of the halides contained in the electrolytes used, while the antioxidative capacity depends on the halides used. In particular, Bi_2_Se_3_ nanosheets passivated using the Cl element have the best stability since they are less prone to air sensitivity. The underlying reason is proposed to be that the larger electronegativity of Cl than that of Br or I leads to an increased electronic distribution from Bi to Cl, resulting in an increased antioxidative capacity for TBAC-Bi_2_Se_3_ devices. This work paves an avenue for adjusting the components of electrolytes to further promote the stability of 2D Bi_2_Se_3_-nanosheet-based electronic devices.

## Figures and Tables

**Table 1:** Atomic ratios of Bi and Se and the oxidized components. The values were estimated from the deconvolved peak areas in Figure 4, and the total number of oxidized and unoxidized Bi atoms in each sample was fixed to 2 for comparison.

	As-Synthesized Bi_2_Se_3_				Air-Exposed Bi_2_Se_3_ for 5 Days			
	Bi 4f	oxid. Bi 4f	Se 3d	oxid. Se 3d	Bi 4f	oxid. Bi 4f	Se 3d	oxid. Se 3d
TBAC-Bi_2_Se_3_	1.66	**0.34**	2.9	0.25	1.48	**0.52**	2.49	0.52
TBAB-Bi_2_Se_3_	1.64	**0.36**	2.85	0.35	0.96	**1.04**	1.91	0.92
TBAI-Bi_2_Se_3_	1.69	**0.31**	2.96	0.34	0.92	**1.08**	1.88	1.08

## Data Availability

The data presented in this study are available on request from the corresponding author.

## Supplementary Materials

The following supporting information can be downloaded at: https://www.mdpi.com/article/10.3390/nano13142056/s1. Figure S1. (a) Linear sweep voltammetry of the electrochemical intercalation of TBA^+^ and (b) the profile of the current change with time at the potential of −2.5 V in the solution containing TBA^+^. Figure S2. Transmission electron microscopy images of the TBAC-Bi_2_Se_3_, TBAB-Bi_2_Se_3_, and TBAI-Bi_2_Se_3_ nanosheets are shown as (a), (b) and (c), respectively. Figure S3: XRD pattern of the Bi_2_Se_3_ powder and PDF#JCPDS:033-0214. The peaks corresponding to the crystal facets were assigned and shown in a dotted line. Nanosheet film was fabricated by spin-coating, while the blue line corresponds to drop casting. Figure S4: (a) Sheet resistance of annealed Bi_2_Se_3_ thin films obtained by different spin-coating times, (b) optical image of spin-coated films on a wafer, and (c) atomic force microscope image of the film with an artificial step. More than 4 times of spin-coating will form a thin Bi_2_Se_3_ film with a sheet resistance of 1 kΩ/sq.
