# Supplementary material for: Antioxidative 2D Bismuth Selenide via Halide Passivation for Enhanced Device Stability
Source: Nanomaterials (Basel). 2023 Jul 12;13(14):2056. doi: 10.3390/nano13142056 (PMC10383381; doi:10.3390/nano13142056)
Supplement: Supplementary file 1 [file nanomaterials-13-02056-s001.zip › nanomaterials-2447862-supplementary.pdf]

# Antioxidative 2D Bismuth Selenide via Halide Passivation for Enhanced Device Stability

**Jiayi Chen [1,2,†], Guodong Wu [1,2,†], Yamei Ding [1,2], Qichao Chen [1,2], Wenya Gao [1,2], Tuo Zhang [1,2], Xu Jing [1,2], Huiwen Lin [1,2], Feng Xue [1,2] and Li Tao [1,2,*]**

[1] School of Materials Science and Engineering, Southeast University, Nanjing 211189, China; jiayi_chen@seu.edu.cn (J.C.); wuguodongjohn@gmail.com (G.W.);

[2] Jiangsu Key Laboratory for Advanced Metallic Materials, Southeast University, Nanjing 211189, China

* Corresponding: tao@seu.edu.cn

† These authors contributed equally to this work.

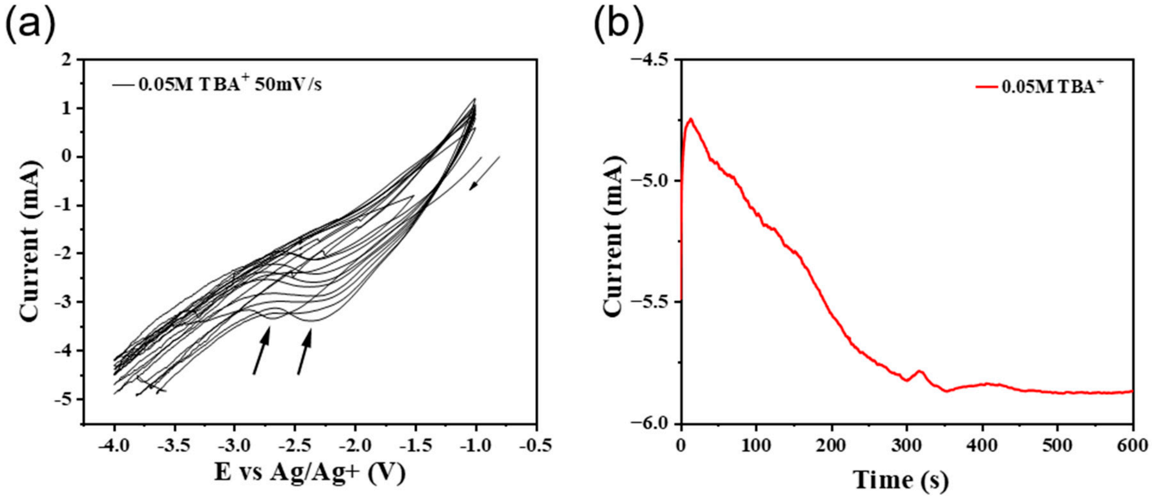


**Figure S1.** (a) Linear sweep voltammetry of the electrochemical intercalation of $TBA^+$ and (b) the profile of the current change with time at the potential of −2.5 V in the solution containing $TBA^+$.

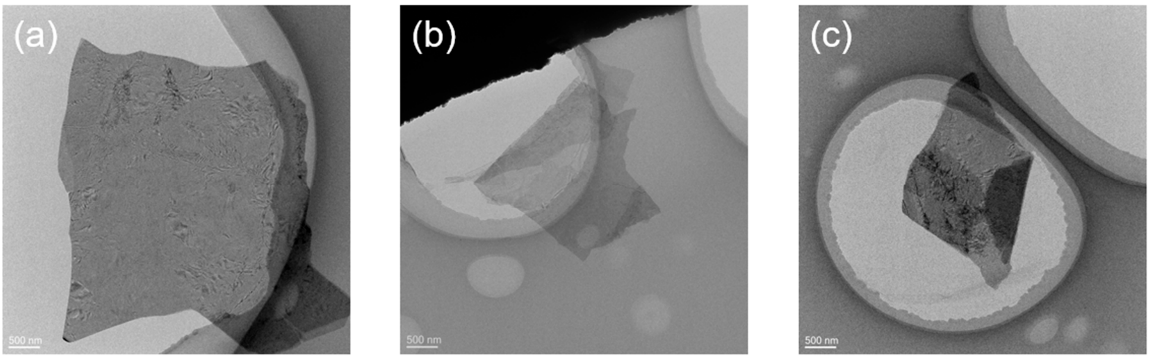


**Figure S2**. Transmission electron microscopy images of the TBAC-$Bi_2Se_3$, TBAB-$Bi_2Se_3$, and TBAI-$Bi_2Se_3$ nanosheets are shown as (a), (b) and (c), respectively.

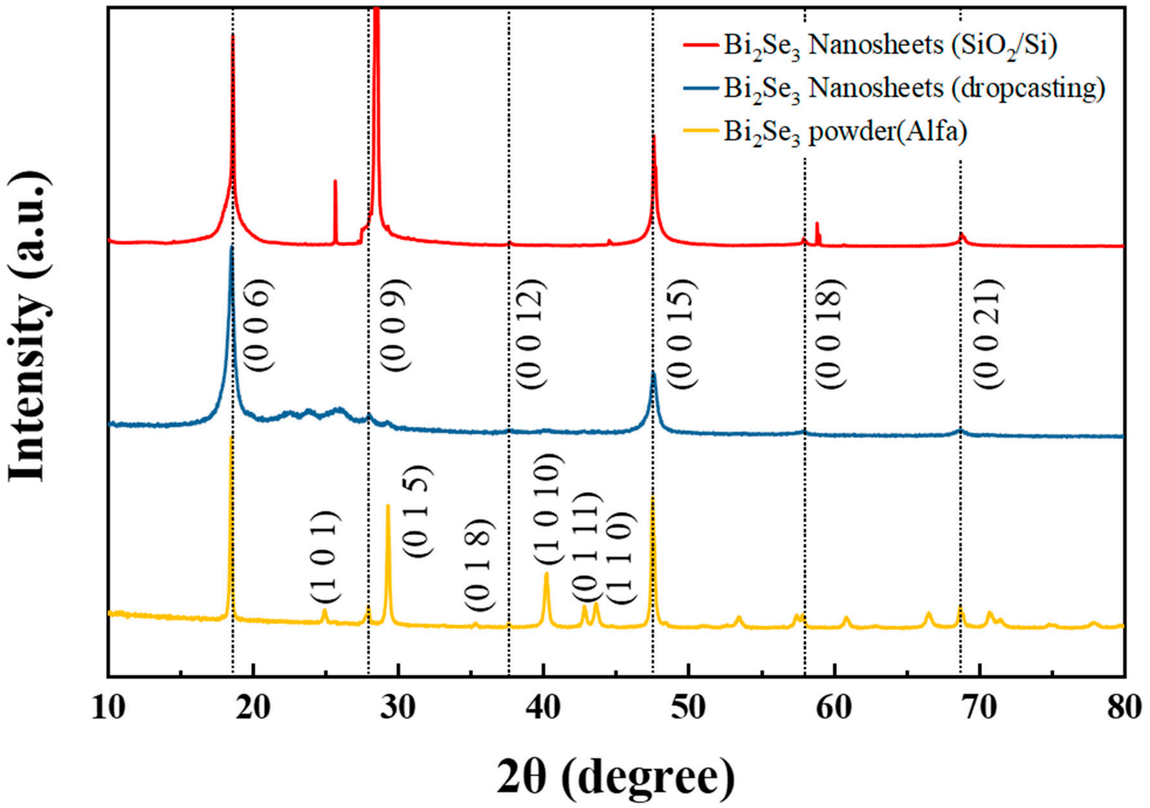


**Figure S3**: XRD pattern of the $Bi_2Se_3$ powder and PDF#JCPDS:033-0214. The peaks corresponding to the crystal facets were assigned and shown in a dotted line. Nanosheet film was fabricated by spin-coating, while the blue line corresponds to drop casting.

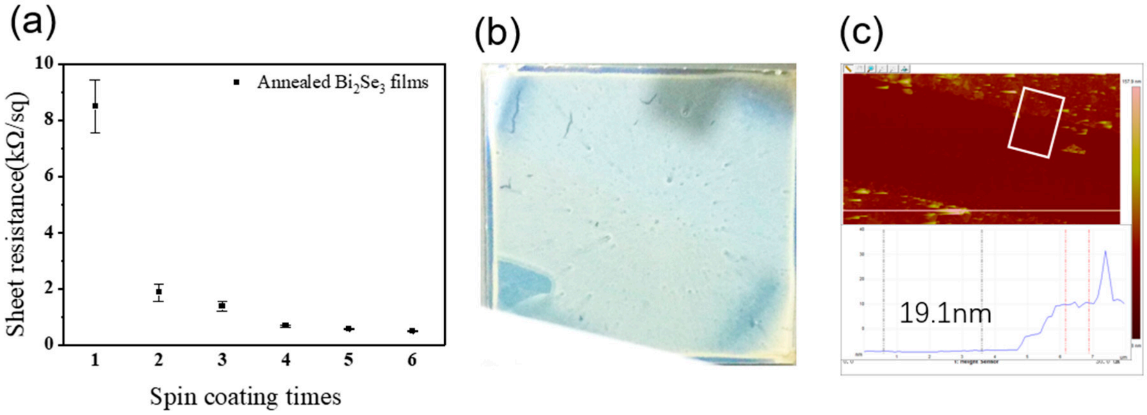


**Figure S4**: (a) Sheet resistance of annealed $Bi_2Se_3$ thin films obtained by different spin-coating times, (b) optical image of spin-coated films on a wafer, and (c) atomic force microscope image of the film with an artificial step. More than 4 times of spin-coating will form a thin $Bi_2Se_3$ film with a sheet resistance of 1 kΩ/sq.
